# Measuring Single-Molecule Conductance at An Ultra-Low Molecular Concentration in Vacuum

**DOI:** 10.3390/mi9060282

**Published:** 2018-06-03

**Authors:** Bo Liu, Makusu Tsutsui, Masateru Taniguchi

**Affiliations:** The Institute of Scientific and Industrial Research, Osaka University, 8-1 Mihogaoka, Ibaraki, Osaka 567-0047, Japan; bo.liu32@sanken.osaka-u.ac.jp (B.L.); taniguti@sanken.osaka-u.ac.jp (M.T.)

**Keywords:** molecular electronics, sensors, contact mechanics, break junction

## Abstract

We report on systematic investigation of single-molecule detection mechanisms in break junction experiments in vacuum. We found molecular feature in the conductance traces at an extremely low concentration of molecules of 10 nM. This was attributed to condensation of the molecular solution on the junction surface upon evaporation of the solvent during evacuation. Furthermore, statistical analyses of the temporal dependence of molecular junction formation probabilities suggested accumulation effects of the contact mechanics to concentrate molecules absorbed on a remote area to the tunneling current sensing zone, which also contributed to the capability of molecular detections at the low concentration condition. The present findings can be used as a useful guide to implement break junction measurements for studying electron and heat transport through single molecules in vacuum.

## 1. Introduction

Substantial research efforts have been devoted to single molecule electronics and related fields since the first current-voltage (I-V) characteristics measurements of benzene-1,4-dithiol [1] using a mechanically controllable break junction (MCBJ). Various fabrication techniques and device structures were realized aiming to fulfill the requirements for the envisaged applications such as molecular field-effect transistors (FETs), diodes, and thermoelectric materials [2,3,4,5,6]. Nowadays, single molecule measurements have become more and more feasible by virtue of break junction methods wherein scanning probe microscope (SPM) set up is usually utilized to address electron transport through metal-molecule-metal structures in organic solvents [3,7]. Meanwhile, from practical viewpoints, such measurements are preferably conducted in dry environments considering that any electronic components would be used in ambient conditions. Despite the circumstance, and the fact that there have been many experimental studies performed in non-liquid environments [7,8,9,10,11], especially those on the thermoelectric properties [12,13], little attention has been paid on the conditions required to examine the single-molecule conductance measurements in vacuum. For instance, 10^−6^ M is often considered to be an appropriate concentration of molecular solution [1,2,3,14,15]. However, what determines the optimal concentration conditions or what will happen if one put too many molecules to the junctions has not been reported. In the present study, therefore, we investigated the details on molecular junction formation mechanisms in break junction experiments using microfabricated MCBJs with emphasis on a lower limit in the molecular concentration to have single-molecule signatures in the break junction measurements. 

## 2. Materials and Methods 

**Figure 1:**
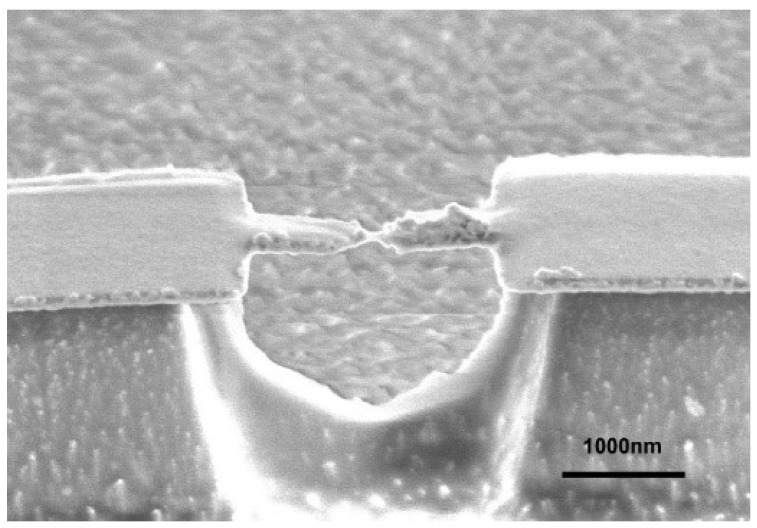
Scanning electron micrograph of a microfabricated mechanically controllable break junction (MCBJ) consisting of a free-standing Au junction on a polyimide-coated phosphor-bronze substrate. Scale bar denotes 1 μm.

Phosphorous bronze beam (42 mm × 5 mm × 0.5 mm) was polished and cleaned by ultrasonication in acetone and isopropanol (IPA). The substrate was coated with a polyimide precursor followed by sequential baking at 120 °C for 1 h, 10 min at 200 °C, and finally 30 min 250 ℃ on a hot plate for the polymerization. The thus formed 10 μm-thick polymer film served as an electrical insulation layer. Microelectrodes were patterned on the polyimide by photolithography with AZ-5206E resist (Integrated Micro Materials, Argyle, TX, USA). After development in NMD-3 (Tokyo Ohka Kogyo, Kawasaki, Japan), a Cr(adhesive)/Au/Cr(adhesive) multilayer of thickness 1 nm/30 nm/1 nm was deposited by radio-frequency magnetron sputtering. The sample was then immersed in *N*, *N*-dimethylformamide (DMF) overnight, which was subsequently ultrasonicated for lift-off of the residual resist. After that, the surface was spin-coated with a positive resist (ZEP520A) and three junctions were delineated by electron beam (EB) lithography on a 100 nm × 100 nm region. The narrowest constriction was designed to be 100 nm wide and 500 nm long. We then deposited 1 nm of Cr adhesion layer with 125 nm thick Au on top. By lifting-off the remnant resist in DMF, three Au junctions were obtained [16]. Finally, the sample was exposed to reactive ion etching with O_2_ etchant gas to remove polyimide underneath the junctions to create 2-µm-long Au nanobridges (Figure 1).

**Figure 2:**
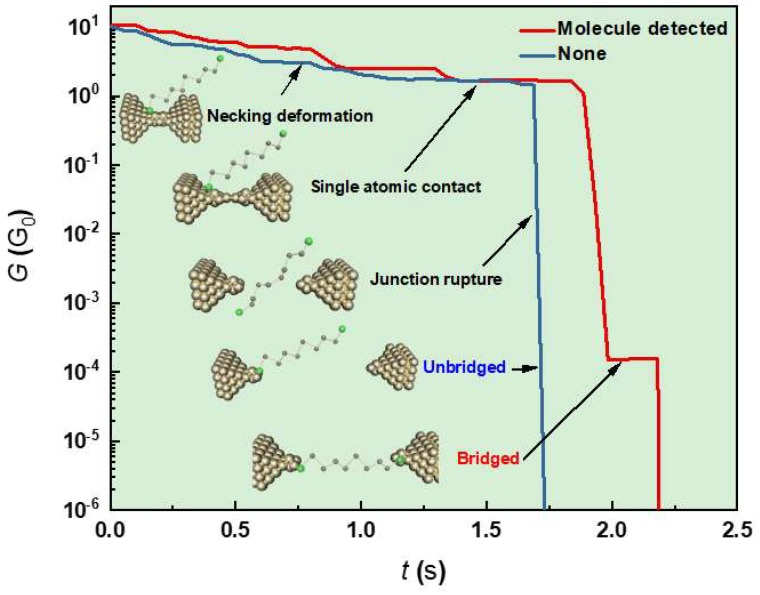
Conductance versus time trace during junction stretching with (red) and without (blue) a 1,8-octanedithiol molecule bridged between the Au electrodes. Inset images explain the contact mechanics involved in the measurement.

In experiments, an MCBJ sample was mounted on a stage in a three-point bending motif: two counter supports hold the sample on the upside, while a push rod is pressing in the center from below (*z*-axis movement) and bending the substrate [17]. Here, the MCBJ set up was designed to enable motion control of the pushing rod with hand by a screw-mechanism as well as computer-control via the piezo-actuator connected to it. On the junction, Teflon cell was bonded with polyimide adhesive. The cell was filled with a dilute solution of target molecules at a specific concentration *c*_ODT_. After that, the substrate bending was examined manually to mechanically break the Au bridge via necking deformation at the narrowest part to let the molecules chemically adsorb on the fresh Au surface (Figure 2). This initial breakage was performed under the applied bias voltage of 200 mV to monitor the junction conductance *G*. By doing so, we could check the junction breakdown via a sudden drop of *G* while carefully stretching the Au junction. After the breakage, the rod was retracted swiftly to quickly reform the junction. Then, the vacuum chamber was evacuated to a pressure below 10^−5^ Torr to remove the organic solvent while remaining the adsorbed molecules on the junction surface. Thereafter, we manipulated the rod position through a LabVIEW-programmed piezo-control (National Instruments, Austin, TX, USA) to repeat Au contact breakdown/formation. Here, special care was taken to manipulate the breaking process by feedback controlling the piezo-motion with respect to *G* in the following manner: (1) The conductance is decreased gradually as the 100 nm-sized junction is thinned by necking deformations at a stretching speed *V_d_* of 2 pm/s. (2) *V_d_* is slowed down to 0.8 pm/s just before the transition from ballistic to tunneling electron transport occurs, whereat the electrical conductance through a monovalent metal contact reaches a level around conductance quantum G_0_ *=* 2*e*^2^*/h*, where *e* is the charge on an electron and *h* is Planck constant. At this point, the Au contact was narrowed to several-atom size. (3) The junction breaks when the substrate is further bent and *G* drops from around 1 G_0_ by orders of magnitude. When the nanogap contained molecular bridges, *G* characteristics display flat plateaus at << 1 G_0_. Otherwise, *G* decreases exponentially with the interelectrode distance as the electrodes are separated with each other at *V_d_*. (4) With over pushing, the junction fully opens by tearing molecule-metal connections apart. After the breakdown, rod is moved in reverse direction to release the bending force to reconnect the electrodes followed by starting of a new trace measurement. 

## 3. Results

1,8-octanedithiol (ODT) was employed as a test molecule whose electrical characteristics have been extensively studied in liquid environments [18,19]. We measured the single-molecule conductance using lithographic MCBJs in vacuum by applying ODT solution of concentration *c*_ODT_ ranging from 1 nM to 1 µM. The volume of solution is controlled by the Teflon cell size into which 2 µL of the sample solution was injected using a pipette. Specifically, we started with 1 nM and collected 1000 traces at room temperature in vacuum. If no molecular signature was observed in the conductance curves, we exhibited the experiment anew, whereat we increased the molecular solution concentration by a factor of 10 each time. These processes were continued on one MCBJ sample until we observed characteristic plateau-like features in the traces.

Below, we show the results obtained when 1,2,4-trichlorobenzene(TCB) was used as solvent to add ODT molecules on Au junction surface. TCB is widely used in SPM break junction measurements in liquid that provides an ultra-clean environment suitable for single-molecule measurements [2,20]. In the solvent, ODT was dissolved at various concentrations from 1 nM to 1 µM. Note that these values do not describe the molecular concentration during the break junction measurements but are the initial conditions to adhere certain amounts of molecules on the junctions. 

**Figure 3:**
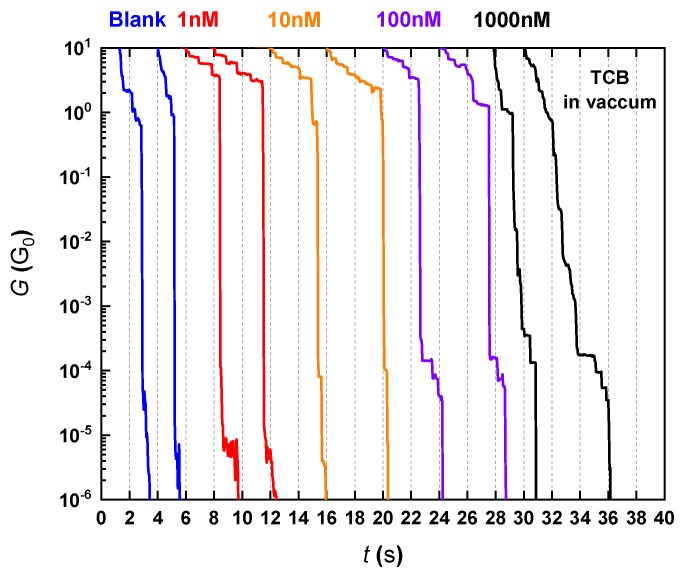
Conductance traces obtained at room temperature in vacuum under various 1,8-octanedithiol (ODT) concentration conditions in 1,2,4-trichlorobenzene (TCB).

Irrespective of absence or presence of ODTs (Figure 3), we observed conductance steps at integer multiples of 1 G_0_ upon elongation of Au junction until a single-atom contact was formed, whereat *G* − *t* traces demonstrated a plateau at ~1 G_0_. Subsequently, the conductance dropped abruptly to *G* < 10^−1^ G_0_. Here, when ODT was not added to TCB, i.e., in a blank test, the conductance often decreased almost linearly in the log*G* − *t* plots from various conductance levels to below 10^−6^ G_0_. This is attributed to exponential decay in the tunneling conductance with the interelectrode distance being steadily extended at constant *V_d_*. On the other hand, in the case of relatively high *c*_ODT_, one or more plateau-like feature appeared occasionally at around 10^−4^ G_0_ indicative of formations of Au-ODT-Au single-molecule junctions [5,21,22,23].

**Figure 4:**
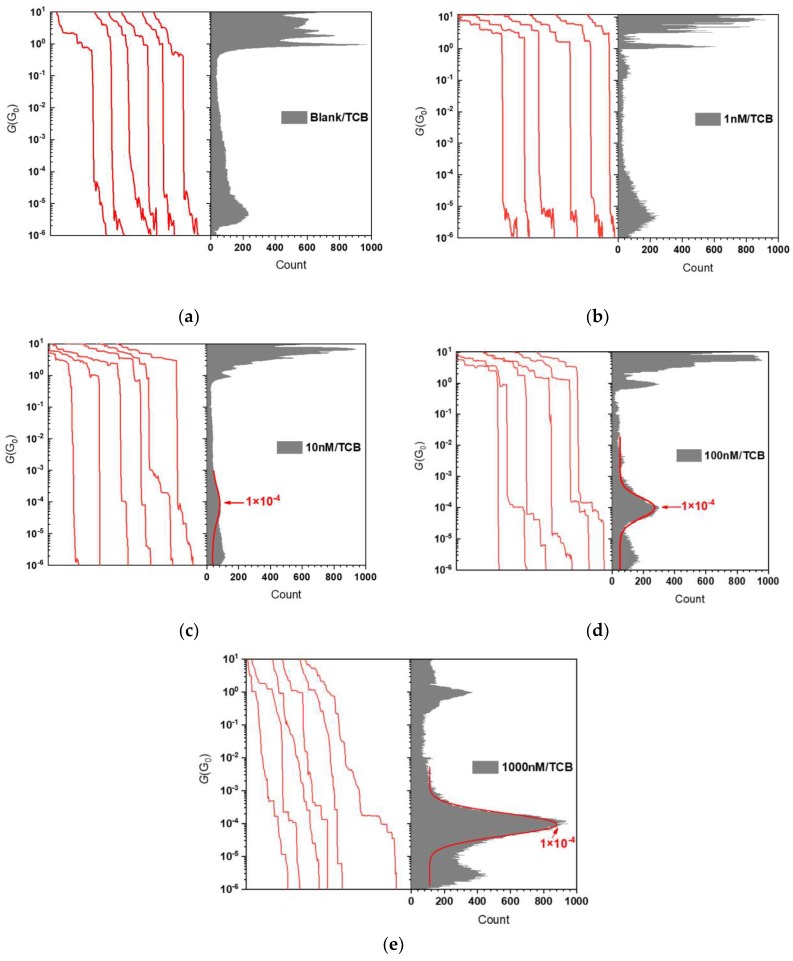
Conductance histograms under various ODT concentrations: (**a**) 0 nM (blank test), (**b**) 1 nM, (**c**) 10 nM, (**d**) 100 nM, and (**e**) 1000 nM. Solid curves are Gaussian fits to the distributions at <10^−3^ G_0_.

We statistically verified whether the initial molecular concentration in the organic solvent affected the characteristics of the molecular signatures found in the log*G* − *t* curves by constructing conductance histograms (Figure 4). At the lowest concentration of 1 nM, no sign of molecular junction formations was confirmed in the traces (Figure 3), wherein they showed exponential decrease in *G* akin to the plots in the blank tests. Accordingly, no conspicuous peaks were observed below 1 G_0_ in the conductance histogram except the one at ~10^−6^ G_0_, which is an artifact stemming from the fact that the contact was opened swiftly when *G* fell below 10^−6^ G_0_.

In sharp contrast to the results at *c*_ODT_ = 1 nM, short conductance plateaus started to appear at approximately 10^−4^ G_0_ when *c*_ODT_ = 10 nM, suggesting temporal trapping of ODT molecules in the Au electrode gap. Further increasing *c*_ODT_ enhanced the probability to observe the molecular feature and also lengthened the plateau. The former result simply indicates higher number of ODTs adsorbed on the Au surface after the evacuation thus increasing the chance to have the dithiol molecules in the gap. The latter effect is presumably a consequence of more frequent formations of multiple Au-ODT-Au bridges in parallel capable of enduring larger amount of strain than single-molecule junctions. Indeed, closer inspections on the conductance plateaus at ~10^−4^ G_0_ revealed larger number of small steps under higher *c*_ODT_ conditions, which can be interpreted as denoting one-by-one fall-downs of the molecular bridges under the tensile forces. Accordingly, as these features both contribute to augment the distribution at 10^−4^ G_0_, the molecular junction conductance peak became more pronounced as *c*_ODT_ increased from 10 nM to 1000 nM (Figure 4).

**Figure 5:**
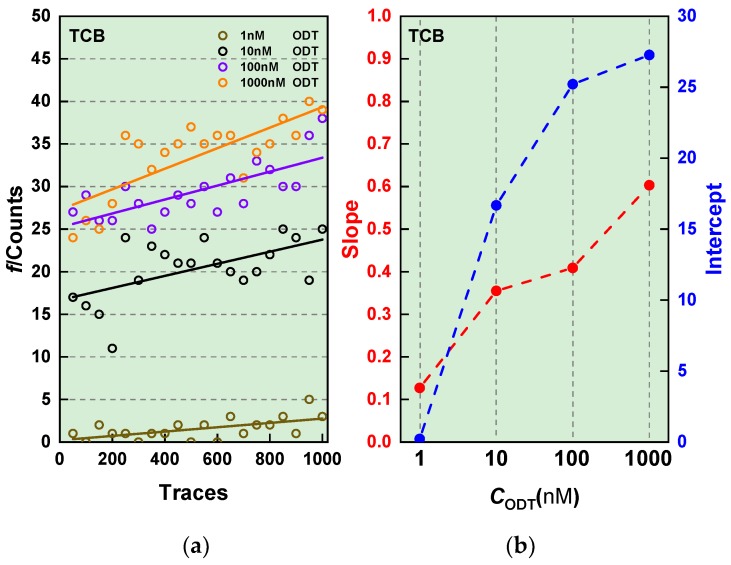
(**a**) Time-course change in the ODT junction formation probability *f*. Solid lines are linear fitting to the plots. (**b**) The slope and intercept of the linear fits in (**a**).

It is surprising that we could detect ODT molecules at 10 nM as there would only be scarce number of molecules per volume, one in every 0.2 μm^3^ of TCB, to have them be trapped in the electrode gap of size as small as 1 nm^3^. To shed light on the underlying mechanism, we analyzed the temporal dependence of the molecular junction formation probability by calculating the frequency *f* of finding the molecular feature defined as plateaus of length longer than 0.5 s in a conductance window of 1 × 10^−4^ G_0_ ± 5 × 10^−5^ G_0_ in every 50 traces from the start of the measurements (Figure 5). Plotting *f* against the number of traces, it became clear that *f* tends to increase not only with *c*_ODT_ but also with time. A possible mechanism for this peculiar behavior is that the repetitive contact formation/breaking processes induce surface migration of the dithiol molecules to move closer to the tips. In fact, Cummings et al. [17] observed denser molecular adsorption on narrower Au junctions in their molecular dynamics simulations. Repeating the deformations would virtually concentrate certain amount of the molecules at the single-molecule sensing zone. This assertion would also explain why we were able to form molecular junctions at the very low *c*_ODT_ conditions.

The above results shed light on the metal-molecule-metal junction formation mechanism in vacuum under the extremely low molecular concentrations. Meanwhile, larger *f* at higher *c*_ODT_ also manifests the importance of the effective concentration of the dithiol molecules on the junction surface. In the present procedure, this concentration is not simply determined by *c*_ODT_ but involves complicated time-dependent condensation processes upon gradual volatilization of the organic solvent. From this point of view, it is anticipated that the minimal *c*_ODT_ to obtain molecular features is dependent on the volatility of the solvent used. Further experiments have therefore been conducted by replacing TCB with Toluene, a more volatile organic solvent. 

**Figure 6:**
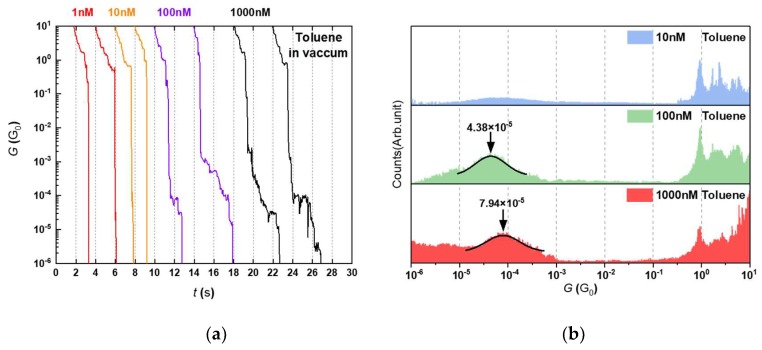
(**a**) Typical conductance traces and (**b**) corresponding histograms in vacuum with ODT dissolved in toluene at various concentration conditions from 1 nM to 1 μM.

**Figure 7:**
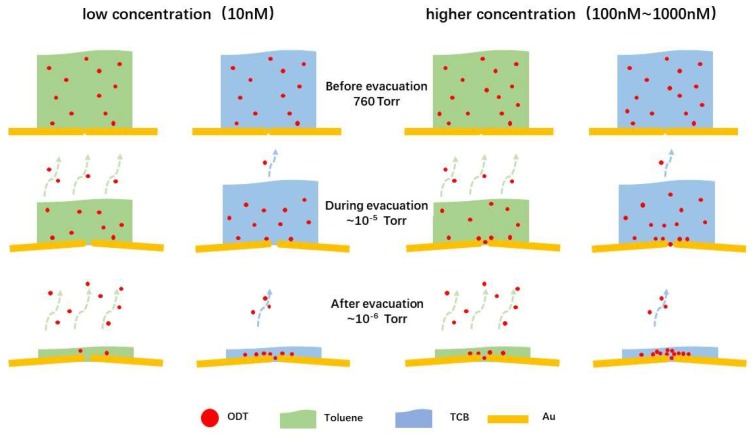
Schematic illustrations depicting solvent dependence of molecular adsorption on Au junctions during evacuation.

Unlike the case of TCB where ODT molecular junctions started to be formed at *c*_ODT_ of 10 nM, there were no molecular features found until the concentration was increased to 100 nM (Figure 6). This means that higher *c*_ODT_ is required to measure the single molecule conductance when using toluene than TCB. The discrepancy would be attributed to solvent properties. The deviation of ODT peaks within one order of magnitude can be a cause of various binding motifs between thiols and gold involved in the break junction experiments with possible adsorption of solvent molecules on Au surface. Compared to TCB gradually volatilizing during evacuation (*P*_0_ = 1 Torr, 25 °C), Toluene tends to evaporate more rapidly (*P*_0_ = 28 Torr, 25 °C). Here, the solute effects on the vapor pressure is negligible due to the vast difference in the amount of ODTs compared to the volume of solvents as predicted by Rault law [24,25],

(1) $$P_{s}=P_{0}\times \frac{n_{Solvent}}{n_{Solvent}+n_{Solute}}$$

where *P*_0_ is the vapor pressure of pure solvent and *n* is amount of substance. As a result, fewer ODTs were able to be accumulated on the surface as they co-evaporate in part with toluene whereby necessitating the high *c*_ODT_ conditions to provide enough amount of dithiol molecules on the Au surface for enabling formation of molecular bridges in the break junction measurements (Figure 7). 

**Figure 8:**
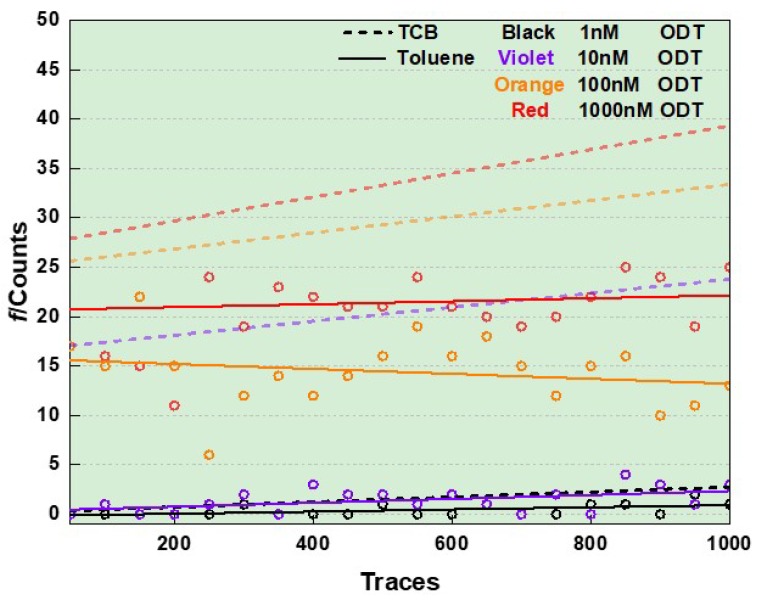
Solvent dependence of the formation probability *f* of ODT junctions. Solid and dashed lines are linear fits to the plots.

We also observed a marked difference in the time-course dependence of the ODT junction formation probability. We plotted *f* as a function of the measurement time denoted by the number of traces for toluene in Figure 8. In case of using TCB as solvent, *f* demonstrated steady increase with *c*_ODT_ manifesting the ODT accumulation effect in the entire concentration conditions tested. In sharp contrast, the trend turns out to be opposite in toluene with *c*_ODT_ = 100 nM suggesting an intrinsic propensity of the repetitive Au contact formation/breakdown to repel the dithiol molecules from the Au tip whereby leading to the gradual decrease in *f* with time. On top of that, the accumulation effect tends to increase *f* with the number of break-junction processes when enough molecules are being added on the junction surface as represented by the plots at 1000 nM having slightly positive slope indicating a weak accumulation effect on *f*.

## 4. Conclusions

We have investigated the molecular bridge formation mechanism in break junction experiments in vacuum using lithographic MCBJs. 1, 8-octanedithiol (ODT) was employed as a model system. The dithiol molecules were introduced via a self-assembly scheme. To evaluate solvent effects on the measurement results, we chose TCB and toluene, which are commonly used in single molecule measurements. Statistical investigations of the conductance traces obtained at various ODT concentrations revealed an important role of surface diffusion in detecting the electron transport properties during the repetitive break junction processes in vacuum that virtually act to concentrate ODTs at remote parts of the junction into the electrode gap regions. Together with the condensation mechanism during vacuum induction that tends to accumulate the molecules on junction surface, this allowed Au-ODT-Au bridge formations and concomitant single-molecule conductance measurements at a critically low molecular concentrations of 10 nM and 100 nM when using TCB and toluene as solvent, respectively. The higher concentration limit in toluene is interpreted as a result of co-evaporation of ODTs. The present finding can be applied to set optimal conditions for implementing single-molecule conductance measurements in vacuum.
